# Severe acute respiratory illness surveillance for influenza in Kenya: Patient characteristics and lessons learnt

**DOI:** 10.1111/irv.12979

**Published:** 2022-03-14

**Authors:** Maryanne N. Gachari, Linus Ndegwa, Gideon O. Emukule, Lily Kirui, Rosalia Kalani, Bonventure Juma, Lilian Mayieka, Peter Kinuthia, Marc‐Alain Widdowson, Sandra S. Chaves

**Affiliations:** ^1^ Kenya Field Epidemiology and Laboratory Training Program (K‐FELTP) Nairobi Kenya; ^2^ Influenza Program Centers for Disease Control and Prevention (CDC) Nairobi Kenya; ^3^ Ministry of Health National Influenza Centre (NIC) Nairobi Kenya; ^4^ Division of Disease Surveillance and Response Ministry of Health Nairobi Kenya; ^5^ Kenya Medical Research Institute (KEMRI) Nairobi Kenya; ^6^ Division of Global Health Protection Centers for Disease Control and Prevention (CDC) Nairobi Kenya; ^7^ Institute of Tropical Medicine Antwerp Belgium; ^8^ Influenza Division Centers for Disease Control and Prevention (CDC) Atlanta Georgia USA

**Keywords:** influenza, influenza hospitalizations, Kenya, respiratory illness, severe acute respiratory illness, surveillance

## Abstract

**Background:**

We describe the epidemiology and clinical features of Kenyan patients hospitalized with laboratory‐confirmed influenza compared with those testing negative and discuss the potential contribution of severe acute respiratory illness (SARI) surveillance in monitoring a broader range of respiratory pathogens.

**Methods:**

We described demographic and clinical characteristics of SARI cases among children (<18 years) and adults, separately. We compared disease severity (clinical features and treatment) of hospitalized influenza positive versus negative cases and explored independent predictors of death among SARI cases using a multivariable logistic regression model.

**Results:**

From January 2014 to December 2018, 11,166 persons were hospitalized with SARI and overall positivity for influenza was ~10%. There were 10,742 (96%) children (<18 years)—median age of 1 year, interquartile range (IQR = 6 months, 2 years). Only 424 (4%) of the SARI cases were adults (≥18 years), with median age of 38 years (IQR 28 years, 52 years). There was no difference in disease severity comparing influenza positive and negative cases among children. Children hospitalized with SARI who had an underlying illness had greater odds of in‐hospital death compared with those without (adjusted odds ratio 2.11 95% CI 1.09–4.07). No further analysis was done among adults due to the small sample size.

**Conclusion:**

Kenya's sentinel surveillance for SARI mainly captures data on younger children. Hospital‐based platforms designed to monitor influenza viruses and associated disease burden may be adapted and expanded to other respiratory viruses to inform public health interventions. Efforts should be made to capture adults as part of routine respiratory surveillance.

## 1 INTRODUCTION

Globally, each year, influenza epidemics result in 3 to 5 million cases of severe illness and approximately 290,000 to 650,000 influenza‐related deaths.^1^ A recent modeling study estimated that the highest burden of annual influenza‐related deaths was in Sub‐Saharan Africa, South East Asia, and Western Pacific regions.^2^ The rate of severe disease is highest in young children <2 years, adults ≥65 years, pregnant women, and individuals with underlying chronic medical conditions.^3^, ^4^ In temperate areas, there are distinct influenza seasons during the cold months, but in the tropics, influenza circulates year‐round, with multiple, often erratic, periods of increased influenza activity,^5^ a pattern also seen in Kenya.^6^

Due to increased globalization, respiratory disease pandemics are likely to spread widely fast, as seen currently with the COVID‐19 pandemic.^7^ In 2006, in response to the pandemic threat of avian influenza A (H5N1), influenza surveillance in Sub‐Saharan Africa was expanded.^8^ Since then, data collected through clinical and virologic surveillance in Africa have contributed to the description of influenza seasonality, characterized genetic make‐up of circulating viruses, and provided viruses for vaccine production.^9^ In Kenya, influenza sentinel surveillance has been ongoing since 2007 (2008) and over the years has evolved to focus exclusively on hospitalized cases. This paper aims to describe the epidemiology and clinical features of patients hospitalized with influenza in Kenya and highlight the importance of year‐round surveillance for severe acute respiratory infections (SARI), especially in tropical countries.

## 2 METHODOLOGY

### 2.1 Study sites

**FIGURE 1:**
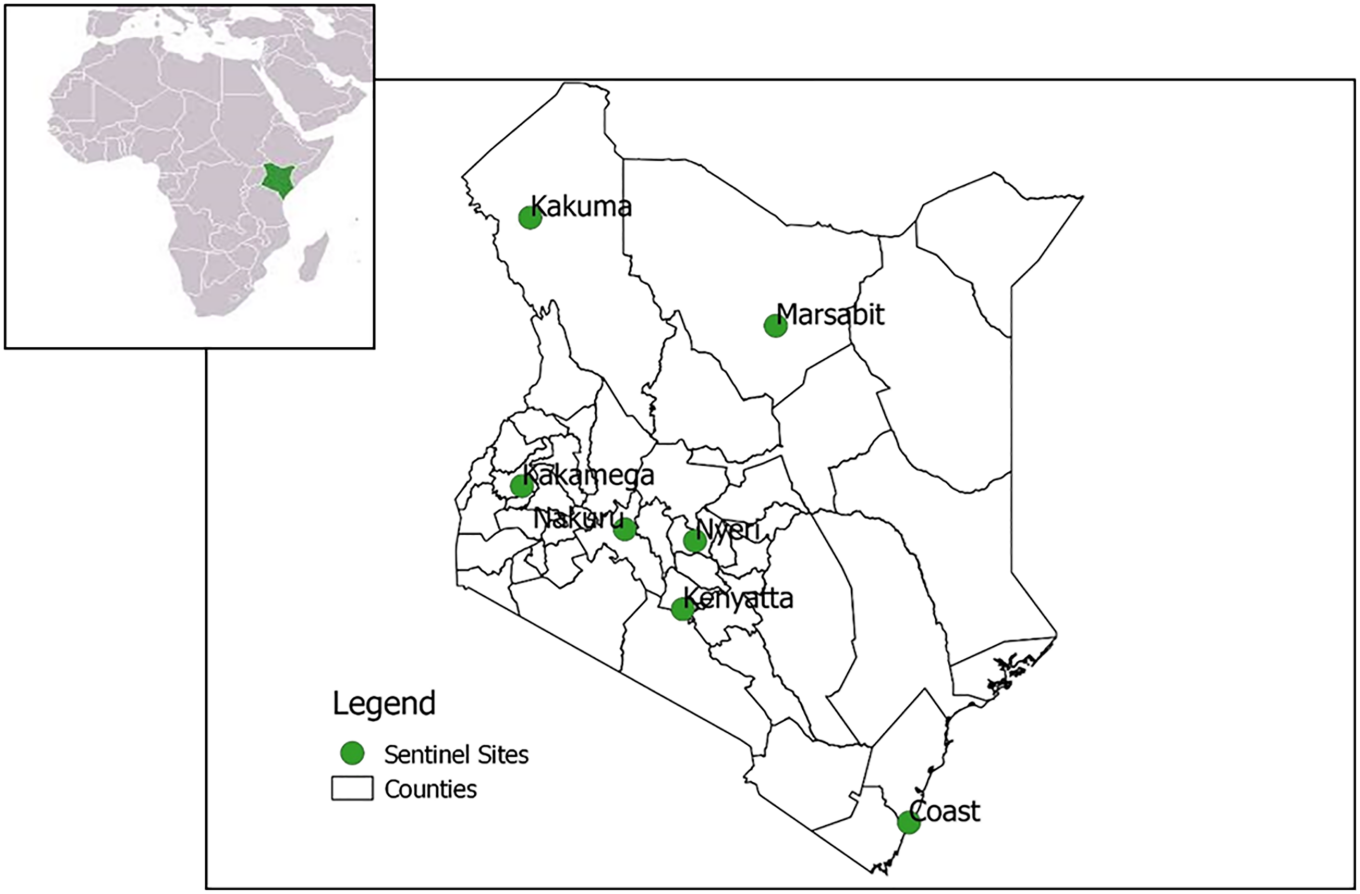
Sentinel surveillance sites for severe acute respiratory illness (SARI) in Kenya

In 2007, the Kenya Ministry of Health (MOH) in collaboration with the US Centers for Disease Control and Prevention, Kenya, (CDC‐K) began sentinel surveillance of influenza in healthcare facilities across the country in order to understand the epidemiology of influenza viruses and detect emerging strains.^10^ In Kenya, current influenza surveillance focuses on SARI hospitalizations in seven sentinel sites: five county referral hospitals (Marsabit County Referral Hospital, Kakamega County General Teaching and Referral Hospital, Nakuru County Referral Hospital, Nyeri County Referral Hospital, and Coast General Teaching and Referral Hospital), Kenyatta National Hospital (tertiary academic referral hospital in Nairobi city), and at the International Rescue Committee (IRC) main hospital in Kakuma refugee camp. In Kenyatta National Hospital, surveillance is carried out in pediatric wards only, while in other sites, patients of all ages are included (Figure 1).

### 2.2 Surveillance procedures

Trained surveillance officers at each surveillance site identified patients with any signs or symptoms of acute respiratory illness by review of inpatient ward admission registers (new admissions on Monday reviewing those admitted during the weekend and Tuesday through Friday to capture new admissions). The surveillance officers approached all potential patients to assess eligibility, specifically if the patient met the case definition for SARI defined as history of fever (or measured fever of ≥38C°) and cough, with onset of symptoms within 10 days prior to hospitalization.

Patients who met the SARI surveillance case definition were approached for verbal consent, and once consent had been obtained, the surveillance officers assigned a unique number to the patient and interviewed either the patient or guardian using an electronic structured questionnaire stored on a password‐protected netbook. Data were collected on demographic and clinical characteristics of patients, including underlying conditions and medical history. A follow‐up exit questionnaire was completed for each patient by abstracting data on clinical outcomes of hospitalization from medical charts upon discharge, death, or transfer. All data were stored on a secure central server maintained by the Kenya Medical and Research Institute (KEMRI). No personal identifiers were stored in the data base.

### 2.3 Specimen collection and shipment

Surveillance officers collected nasopharyngeal (NP) or nasal (NS) and/or oropharyngeal (OP) swabs from all SARI patients within 48 h of hospital admission during Monday–Wednesday and only the clinical and risk‐factor data (without specimens) on Thursdays and Fridays. NP and OP swabs were collected during the period 2014 through 2016 and NS and OP swabs between 2017 and 2018. A dacron‐tipped flexible aluminum‐shafted swab was inserted through the nose into the posterior nasopharynx (or, if nasal swab, up to the nostril less than 1 inch [about 2 cm] or until you feel resistance), where it was rotated 180° and left in place for 3–5 s. A separate swab was inserted into the oropharynx and swabbed over the lower portion of the oropharynx. The two swabs were placed into a freshly prepared single vial containing viral transport media labeled with the patient's unique identifier, barcode number, and date of collection. Samples were maintained at 2–8°C in the laboratory of each participating medical facility for up to 36 h and then packaged and shipped to the National Influenza Center (NIC) Laboratory, in Nairobi, for testing every Thursday of the week.

### 2.4 Specimen processing

An aliquot of each specimen was tested by real‐time reverse transcription polymerase chain reaction (rtRT‐PCR) for influenza virus types A and B at the NIC, using protocol, primers, and probes provided by the Influenza Division, CDC‐Atlanta, USA, as previously described.^10^ Briefly, total ribonucleic acid (RNA) was extracted from 140 mL aliquots of each specimen using a QIAamp viral RNA mini kit (Qiagen GmbH, Germany) according to the manufacturer's instructions. One‐step rtRT‐PCR was carried out using the AgPath kit (Applied Biosystems, California, USA). Values with a cyclic threshold (*C*_*T*_) reading <40 were recorded as positive. All specimens positive for influenza type A were subtyped for H3 and pH1N1 using rtRT‐PCR. Specimens that were positive for influenza A virus by rtRT‐PCR but failed to subtype were sent to the WHO Influenza Collaborating Center at CDC‐Atlanta, GA, USA, for further antigenic characterization.

### 2.5 Data management and analysis

Data analysis was performed by epidemiologists at the Division of Disease Surveillance and Response (DDSR) and the NIC at the MOH, KEMRI, and CDC‐Kenya. Weekly reports of aggregated clinical and laboratory data were generated and shared with stakeholders including the World Health Organization (WHO) and the national Public Health Emergency Operations Center at the Kenya MOH for situational awareness. For this manuscript, we downloaded data from January 2014 to December 2018 into Microsoft Excel (Microsoft Office, Seattle, USA, 2013), cleaned, and uploaded into Epi info 7.2.3.0 (CDC, Atlanta, 2019) and STATA 14.2 (College Station, Texas 77 845 USA, 2018) for analysis.

Because children and adults would likely differ in risk factors and severity, the analysis was done separately for children (<18 years) and adults. We used proportions to describe the demographic and clinical characteristics of SARI cases separately for adults and children. We compared influenza positive cases with those testing influenza negative, for children only due to limited sample size among adults, using Chi‐square test statistic (or Fisher's exact test if applicable) for various demographic, clinical characteristics, and outcomes. Children with no recorded laboratory result were excluded from this analysis. We assessed the severity of disease among children by comparing clinical and treatment characteristics (tachypnea, hypoxia, high fever, intravenous fluids and blood transfusion treatment, admission to intensive care unit [ICU], and death) among influenza positive cases and those testing negative. We used graphs to display trends of influenza positivity for hospitalized children with SARI who were tested for influenza in the 5‐year period and showed the types and subtypes detected. Those who had absconded care or refused treatment were excluded as their outcome status could not be confirmed. We then pre‐selected variables that were potential confounders (age, sex, length of time from illness onset to hospitalization, and any chronic illness) or with a *p* value of <0.2 to include in a multivariable logistic regression model to assess predictors of death. Variables that had a *p* value of <0.05 in the final model were considered statistically significant.

### 2.6 Ethical considerations

Data from this surveillance were regarded by the Kenya MOH as a routine public health activity and received a non‐research project determination by CDC and thus did not require an ethical review. Verbal consent was obtained from all participants before administration of questionnaires and collection of specimens. For children, verbal consent was obtained from the parent/guardian and assent for children ≥7 years.

## 3 RESULTS

### 3.1 Demographic and clinical characteristics of children and adults hospitalized with severe acute respiratory illness

From 2014 through 2018, 11,166 persons hospitalized with SARI were identified in the seven surveillance sites. Among them there were 10,742 (96%) children aged <18 years and 424 (4%) adults. The median age of children was 1 year (IQR = 6 months, 2 years), 5711 (53.2%) were aged 6–23 months, and 5979 (55.6%) were males. Hospitalizations associated with SARI among adults mostly represented people 18–64 years (85%), and 61.8% of all adults hospitalized were males. The median age among adults was 38 years (IQR = 28 years, 52 years). Underlying medical conditions were present among 2142 (19.9%) children and 214 (50.4%) among adult SARI cases. The overall influenza positivity among samples tested from two thirds of children and adults was comparable (10.0% and 11.7%, respectively) (Table 1). There were 716 (10.0%) children with laboratory confirmed influenza. Because the number of adult cases was limited, the rest of the analysis was focused only on children from this point forward.

**TABLE 1:** Characteristics among children and adults hospitalized with severe acute respiratory illness, 2014–2018, Kenya

Characteristic	Children *N* = 10 742	Adults *N* = 424
	*n* (%)	*n* (%)
Sex		
Male	5976 (55.6)	262 (61.8)
Female	4766 (44.4)	162 (38.2)
Age group		
<6 months	2185 (20.3)	–
6–23 months	5711 (53.2)	–
2–<5 years	2169 (20.2)	–
5–17 years	677 (6.3)	–
18–64 years	–	361 (85.1)
≥65 years	–	63 (14.9)
Site		
Kakamega	1363 (12.7)	69 (16.3)
Kakuma	1330 (12.4)	26 (6.1)
Kenyatta National Hospital^a^	1600 (14.9)	0
Marsabit^b^	81 (0.8)	2 (0.5)
Mombasa	1962 (18.3)	2 (0.5)
Nakuru	2686 (25.0)	266 (62.7)
Nyeri	1720 (16.0)	59 (13.9)
Year		
2014	1237 (11.5)	68 (16.0)
2015	2005 (18.7)	184 (43.4)
2016	2885 (26.9)	121 (28.5)
2017	1752 (16.3)	19 (4.5)
2018	2863 (26.7)	32 (7.6)
Presence of any underlying medical condition^c^	2142 (19.9)	214 (50.4)
Median time from illness onset to hospital admission in days (IQR)	3 days (IQR = 1, 4)	3 days, (IQR = 2, 6)
Median length of hospital stay (IQR)	3 days (IQR = 2, 8)	4 days (IQR = 2, 8)
Respiratory sample collected^d^	7175 (66.8)	273 (64.4)
Laboratory‐confirmed influenza	716 (10.0)	32 (11.7)

Abbreviation: IQR, interquartile range.

^a^ Surveillance includes only pediatric cases aged <12 years.

^b^ Site added in 2017.

^c^ Includes the following: chronic respiratory illness, chronic neuromuscular or neurological disease, newly diagnosed tuberculosis, HIV/AIDS, chronic cardiac, liver or renal disease, malnutrition, diabetes, asthma or cancer, sickle cell disease, and rickets.

^d^ Percentage of persons with respiratory sample collected.

### 3.2 Comparison of children with SARI among those with and those without lab‐confirmed influenza

When comparing children with influenza and those without, there were significant differences observed by age group (*p* < 0.001); the <6 months age group was underrepresented among influenza positive cases compared with influenza negative (9.5% vs. 21.1%). Nonetheless, most clinical outcomes and severity were similar, with a small difference in the time from disease onset to hospitalization where influenza negative cases seem to be hospitalized earlier in the course of the disease compared with influenza positive cases (68.8% vs. 63.3%, respectively; *p* value <0.001), and a lower frequency of tachypnea was reported among the influenza positive cases (36.0% vs. 43.8%; *p* < 0.001) (Table 2).

**TABLE 2:** Comparison of demographic and clinical characteristics of laboratory‐confirmed influenza positive and negative severe acute respiratory illness (SARI) associated hospitalizations among children, 2014–2018, Kenya

Characteristic	Influenza positive	Influenza negative	*P* value
	*N* = 716	*N* = 6287	
	
Age group	*n* (%)	*n* (%)	
< 6 months	68 (9.5)	1326 (21.1)	
6–23 months	357 (49.9)	3344 (53.2)	
2–<5 years	224 (31.3)	1266 (20.1)	
5–17 years	67 (9.4)	351 (5.6)	<0.001
Sex			
Female	323 (45.1)	2758 (43.9)	
Male	393 (54.9)	3529 (56.1)	0.55
Time from illness onset to hospital admission			
0–3 days	446/705 (63.3)	4261/6196 (68.8)	
4–7 days	217 (30.8)	1670 (27.0)	
8 days	42 (6.0)	265 (4.3)	0.001
Median duration of time from illness onset to admission (IQR)	3 days (IQR = 1, 5)	2 days (IQR = 1, 4)	
Length of hospital stay			
0–3 days	253/679 (37.3)	2188/5773 (37.9)	
4–7 days	251/679 (37.0)	2125 (36.8)	
> 7 days	175/679 (25.8)	1460 (25.3)	0.78
Median length of hospital stay (IQR)	5 days (IQR = 3, 8)	4 days (IQR = 3,8)	
Comorbidities (yes vs. no)			
Presence of any underlying illness^a^	139 (19.4)	1250 (19.9)	0.809
HIV positive^b^	2/152 (1.3)	19/988 (1.9)	0.846
Malaria positive^c^	32/146 (21.9)	222/1130 (19.6)	0.591
Tuberculosis treatment	5/706 (0.7)	79/6145 (1.3)	0.254
Severity of cases (yes vs. no)			
Tachypnea^d^	257/714 (36.0)	2744/6260 (43.8)	<0.001
Hypoxia^e^	71/687 (10.3)	597/5966 (10.0)	0.838
High fever^f^	271/715 (37.9)	2335/6280 (37.2)	0.736
Received intravenous fluids	71/295 (24.1)	577/2082 (27.7)	0.213
Received blood transfusion	15/295 (5.1)	103/2082 (4.9)	1.000
Admitted to intensive care unit	61/707 (8.6)	449/6148 (7.3)	0.232
Discharge diagnosis			
Malaria	35/295 (11.9)	239/2082 (11.5)	0.923
Pneumonia	216 (73.2)	1564 (75.1)	0.527
Sepsis	11 (3.7)	107 (5.1)	0.368
Meningitis/encephalitis	24 (8.1)	145 (6.9)	0.540
Dehydration	22 (7.5)	194 (9.3)	0.351
Malnutrition	40 (13.6)	288 (13.8)	0.970
Bronchitis	15 (5.1)	125 (6.0)	0.620
Asthma exacerbation	4 (1.4)	33 (1.6)	0.963
Acute respiratory distress syndrome	3 (1.0)	10 (0.5)	0.455
Tuberculosis	7 (2.4)	39 (1.9)	0.543
Gastroenteritis	36 (12.2)	347 (16.6)	0.065
Anemia	24 (8.1)	177 (8.5)	0.921
Outcome			
Death	23/701 (3.3)	217/6093 (3.6)	
Discharged alive	678/ (96.7)	5876 (96.4)	0.785

Abbreviation: IQR, interquartile range.

^a^ Includes the following: chronic respiratory illness, chronic neuromuscular or neurological disease, newly diagnosed tuberculosis, HIV/AIDS, chronic cardiac, liver or renal disease, malnutrition, diabetes, asthma or cancer, sickle cell disease, and rickets.

^b^ Number of positive HIV cases out of 1140 SARI cases tested during hospitalization.

^c^ Number of malaria positive cases out of 1276 SARI cases tested during hospitalization.

^d^ Tachypnea defined as <2 months: >60 breaths/min; 2 months to <1 year: >50 breaths/min; 1 year to <5 years: >40 breaths/min; ≥5 years: >20 breaths/min.

^e^ <90% oxygen saturation.

^f^ >38.0°C.

**FIGURE 2:**
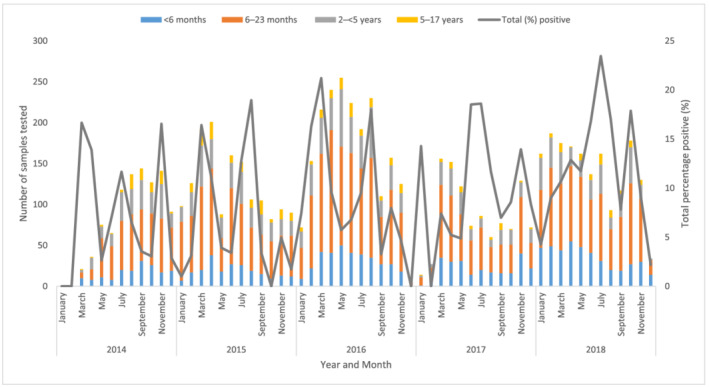
Frequency of severe acute respiratory illness associated hospitalizations among children tested for influenza and the percentage of positivity by month and age group, 2014–2018, Kenya (*N* = 7175)

Overall, the median influenza positivity rate of SARI samples tested for the entire period was 7% (IQR = 3.6%–13.9%), with the highest positivity rate of 23% detected in July 2018. There was not any clear pattern of seasonality observed (Figure 2). Overall, highest percentage of influenza positivity was in those aged 5–17 years (16%; 95% CI 12.6, 19.5) and 2–4 years (15%; 95% CI 13.0, 16.6). The temporal distribution of the types and subtypes of influenza viruses among children that were detected during the study period is shown in (supporting information Figure S1).

### 3.3 Comparison of case fatality proportion (CFP) among children with and those without lab‐confirmed influenza

**FIGURE 3:**
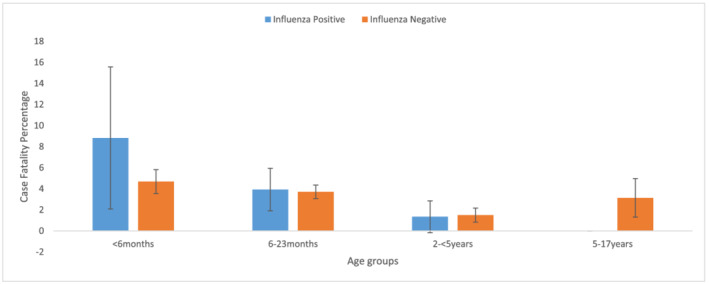
Case fatality percentages of laboratory‐confirmed and influenza negative hospitalized children with respective confidence intervals, by age group, 2014–2018, Kenya

Overall, there was no significant difference in the CFP between hospitalized children who tested positive for influenza (3.3%, 95% CI 1.9, 4.5) and those who tested negative (3.6%, 95% CI 3.2, 4.2). Young children aged less than 6 months who were influenza positive had a higher but not statistically significant CFP of 8.8% (95% CI 2.1, 15.6) compared with 4.7% (95% CI 4.7 3.5, 5.8) among those who tested negative (Figure 3). No notable differences were observed among older children (6–23 months and 2–4 years). Influenza positive SARI cases who were hospitalized >7 days after illness onset had a higher but not statistically significant CFP of 11.9% (95% CI 3.9, 25.6) compared with 2.9% (95% CI 1.6, 4.9) among those hospitalized early (<4 days from illness onset). Tuberculosis significantly increased CFP in both influenza positive and negative groups: 20% (95% CI 0.5, 71.6) for those influenza positive and 16.5% (95% CI 9.1, 26.5) for those influenza negative. Influenza negative SARI cases with a reported underlying illness had higher CFP 6.9% (5.5, 8.4) compared with 4.3% (1.6, 9.2) among influenza positive SARI cases (supporting information Table S1).

### 3.4 Factors associated with in‐hospital death among hospitalized children with SARI

The final model accounted for age group, sex, time from illness onset to hospitalization, and presence of any comorbidity. In the multivariable model, children with any reported underlying illness (including TB, HIV/AIDS, malnutrition, diabetes, asthma, cancer, chronic cardiac, and liver or renal disease) had greater odds of dying in the hospital compared with those who did not have any (aOR 2.11, 95% CI 1.09, 4.07). Children who tested positive for HIV during admission had greater odds of dying in the hospital compared with those who tested negative (aOR 9.25, 95% CI 3.17, 26.96) (Table 3).

**TABLE 3:** Predictors of in‐hospital death among children admitted with severe acute respiratory illness 2014–2018, Kenya

Characteristic	Death, *N* = 384	Discharged alive, *N* = 10,048	Crude odds ratio (95%CI)	*P* value	Adjusted odds ratio (95%CI)	*P* value
Age group	*n* (%)	*n* (%)				
<6 months	110 (28.6)	2019 (20.1)	1.89 (1.14–3.14)	0.015	1.27 (0.43–3.77)	0.67
6–23 months	225 (58.6)	5352 (53.3)	1.46 (0.89–2.38)	0.15	0.7 (0.25–1.95)	0.49
2–<5 years	31 (8.1)	2050 (20.4)	0.52 (0.29–0.94)	0.045	0.47 (0.14–1.63)	0.24
5–17 years	18 (4.7)	627 (6.2)	**Ref**		**Ref**	
Sex						
Female	171 (44.5)	4445 (44.2)	1.0 (0.82–1.24)	0.95	0.54 (0.28–1.03)	0.06
Male	213 (55.5)	5603 (55.8)	**Ref**		**Ref**	
Time from illness onset to hospitalization						
0–3	210/383 (54.8)	6930/9954 (69.6)	**Ref**		**Ref**	
4–7	135 (35.2)	2597 (26.1)	1.72 (1.38–2.14)	<0.001	1.48 (0.76–2.89)	0.25
> 7	38 (9.9)	427 (4.3)	2.90 (2.05–4.20)	<0.001	3.70 (0.82–16.58)	0.09
Comorbidities						
Any underlying illness^a^	142 (37.0)	1922/9924 (19.4)	2.44 (1.97–3.02)	<0.001	2.11 (1.09–4.07)	0.03
HIV positive^b^	5/44 (11.4)	24/1939 (1.2)	10.2 (3.7–28.2)	<0.001	9.25 (3.17–26.96)	<0.001
Malaria positive^c^	9/47 (19.1)	498/2369 (21.0)	0.89 (0.43–1.85)	0.90		
Tuberculosis treatment	15/382 (3.9)	139/10,030 (1.4)	2.91 (1.69–5.00)	<0.001	1.08 (0.23–5.03)	0.91
Laboratory‐confirmed influenza	23/240 (9.6)	678/6554 (10.3)	0.92 (0.59–1.42)	0.78		

Abbreviations: IQR, interquartile range; SARI, severe acute respiratory illness.

^a^ Includes the following: chronic respiratory illness, chronic neuromuscular or neurological disease, newly diagnosed TB, HIV/AIDS, chronic cardiac, liver or renal disease, malnutrition, diabetes, asthma or cancer, sickle cell disease, and rickets.

^b^ Number of positive HIV cases out of 1150 SARI cases tested during hospitalization.

^c^ Number of malaria positive cases out of 1282 SARI cases tested during hospitalization.

## 4 DISCUSSION

We analyzed data from patients hospitalized with SARI from countrywide sentinel sites to describe the epidemiology of circulating influenza viruses in Kenya. However, the majority (96%) of hospitalized SARI cases were children aged <18 years, while only 4% were adults. Among the hospitalized adults, 15% were older adults aged ≥65 years, an age group recognized as being at increased risk for influenza complications and death.^11^ Among children with a reported underlying illness, in‐hospital death was twice as likely to occur compared with those who did not have any reported. Nonetheless, the data do not show a particularly high risk of death among children with influenza compared with those without. Overall, 10% of children hospitalized with SARI had laboratory‐confirmed influenza, with the peak percentage positivity among school age children (aged 5–17 years). SARI surveillance may need to encompass monitoring of other respiratory pathogens that are responsible for healthcare utilization and disease burden to optimize resources.

The surveillance system in Kenya mainly captured children aged <5 years, representing 94% of all SARI cases. In public health facilities countrywide, hospital user fees for children aged <5 years are subsidized by the government, and as a result, young children may be overrepresented in this surveillance due to differentiated care seeking.^12^ Adults may be more likely to delay healthcare seeking,^13^ and this may result in reduced likelihood of influenza virus detection in this population. The potential delay or refrain in using the healthcare system among adults might have contributed to worse clinical outcomes due to a late clinical intervention, although this could not be assessed in this platform. Moreover, SARI case definitions may not capture older hospitalized patients with laboratory‐confirmed influenza who present with atypical signs and symptoms which are not included in standard SARI case definition.^14^ Inclusion of private hospitals as sentinel sites may make the system more representative of adult population as subsidized care would have less of an impact on healthcare utilization.

Approximately one tenth of children hospitalized with SARI tested positive for influenza, similar to results from other surveillance sites in Sub‐Saharan Africa like Rwanda (6.3%),^15^ Zambia (5.5%),^16^ Malawi (11.3%),^17^ and South Africa (7.3%).^18^ This suggests that there is a high rate of other respiratory illness etiologies that could explain some of the hospitalizations associated with cough and fever. Broader testing could allow for identification of common and emerging respiratory pathogens as well as monitoring the impact of public health interventions. During the COVID‐19 pandemic, the World Health Organization leveraged influenza surveillance networks and tools to monitor COVID‐19.^19^ Capitalizing on existing resources can make surveillance networks more sustainable and robust. Local and national governments as well as funders (from the private and public sector) may want to consider broadening objectives of the SARI surveillance by testing samples for a wider range of pathogens and whether adapted case definitions could be used to encompass a larger variety of respiratory pathogen disease presentations. The overall CFP among influenza‐associated SARI cases was 3.3%, an increase from the reported CFP of 0.9% in the previous 5 years of surveillance in this system.^10^ This is comparable with reported CFPs from studies done in South Africa (3%)^20^ and Egypt (2%).^21^ The overall CFP in influenza‐associated SARI patients and those who were influenza negative was comparable (3.3% vs. 3.6%). Previously, in Sub‐Saharan Africa, among hospitalized patients with SARI, the CFP among influenza‐associated SARI cases was described as lower than in those who are influenza negative (1.8% vs. 2.9%),^22^ but this could have been influenced by lack of systematic data collection on influenza‐associated outcomes among countries participating in that study. The CFP reported in infants aged <6 months with laboratory‐confirmed influenza was high, highlighting the importance of vaccinating pregnant women with influenza vaccine to protect newborn infants from influenza virus infection.^23^ Higher CFPs were also identified in SARI cases (with or without influenza) with a diagnosis of tuberculosis. In addition, children with reported underlying illness, especially those living with HIV, had greater odds of in‐hospital death compared with HIV negative children. In countries with high HIV prevalence in Africa, persons living with HIV^24^ and those with chronic lung conditions such as tuberculosis are at risk of influenza‐associated severe disease.^25^, ^26^

The highest influenza percentage positivity in our surveillance data was reported in school aged children 5–17 years, who are known to shed influenza virus for longer periods and to increase community transmission.^27^ School‐based influenza vaccination programs recommended in the United States and United Kingdom have significant benefits because this reduces transmission to vulnerable older age groups not easy to reach as part of the healthcare system.^28^, ^29^ The burden of healthcare utilization associated with school aged children may need to be taken into account as the Kenya government considers recommendations for the use of influenza vaccines.

Our study had some limitations. We did not have information on the total number of hospital admissions to determine the burden of influenza and SARI hospitalizations in the context of overall hospital admissions. Estimates of disease burden using hospital data in this study should be interpreted cautiously, because in Kenya, there is limited access to care due to economic and logistic reasons. Previously, studies have shown that in countrywide, only 20% of influenza‐associated SARI cases are hospitalized^30^ even though many severe cases occur in the community. Moreover, the surveillance system captured a small proportion of older patients; therefore, counting influenza cases and associated deaths in the hospital setting may lead to underestimation of disease burden. In this surveillance, we only tested samples for influenza viruses and a large proportion of SARI cases were likely associated with non‐influenza etiology which remains unknown. Expanded testing would be helpful to understand the etiology of severe acute respiratory disease in Kenya.

## 5 CONCLUSION

We found that the surveillance system mainly captured children aged <5 years. Hospitalized children with SARI who had a reported underlying illness had higher likelihood of in‐hospital death compared with those without. A high influenza‐associated CFP was reported among infants aged <6 months. These groups are disproportionately affected and may be priority targets for influenza control programs. With the emergence of new respiratory pathogens and the development of new vaccines targeting respiratory illnesses such as the COVID‐19 vaccine, hospital‐based platforms designed to monitor influenza viruses and associated disease burden should consider adapting their tools and expanding surveillance to capture systematically respiratory viruses that can inform public health interventions and future investments in respiratory disease prevention.

## AUTHOR CONTRIBUTIONS

**Linus Ndegwa:** Conceptualization; formal analysis; methodology. **Gideon O. Emukule:** Conceptualization; formal analysis; methodology. **Lily Kirui:** Data curation. **Rosalia Kalani:** Data curation. **Bonventure Juma:** Data curation. **Lilian Mayieka:** Data curation. **Peter Kinuthia:** Data curation. **Marc‐Alain Widdowson:** Conceptualization; formal analysis; methodology. **Sandra S Chaves:** Conceptualization; formal analysis; methodology.

## CONFLICT OF INTEREST

The authors have no conflict of interest to disclose.

## DISCLOSURE

The findings and conclusions in this report are those of the authors and do not necessarily represent the official position of the Centers for Disease Control and Prevention and the Kenya Ministry of Health (MOH).

### PEER REVIEW

The peer review history for this article is available at https://publons.com/publon/10.1111/irv.12979.

## Supporting information




**Figure S1.** Distribution of influenza virus types and subtypes detected among children with severe acute respiratory illness by month, 2014–2018, Kenya (N = 716)

**Table S1.** Case Fatality Percentages (CFP) of children admitted with severe respiratory acute illness (SARI), 2014–2018, Kenya

## Data Availability

Some access restrictions apply to the data underlying the findings in this manuscript. For ethical reasons we cannot publish the data sets on line, however requests for the data underlying the findings presented in our manuscript can be made to the Kenya Ministry of Health through Dr. Daniel Langat, Head of Division of Disease Surveillance and Response, Email: langatdoc@gmail.com.

## References

[1] WHO . Influenza (seasonal).int/en/news. https://www.who-room/fact-sheets,detail/influenza-(seasonal) (2018).

[2] Iuliano AD , Roguski KM , Chang HH , et al. Estimates of global seasonal influenza‐associated respiratory mortality: A modelling study. Lancet. 2019;391(10127):1285‐1300. 10.1016/S0140-6736(17)33293-2 PMC5935243 29248255

[3] Gill PJ , Ashdown HF , Wang K , et al. Identification of children at risk of influenza‐related complications in primary and ambulatory care: A systematic review and meta‐analysis. Lancet Respir Med. 2015;3(2):139‐149. 25481379 10.1016/S2213-2600(14)70252-8

[4] Meier CR , Napalkov PN , Wegmüller Y , Jefferson T , Jick H . Population‐based study on incidence, risk factors, clinical complications and drug utilisation associated with influenza in the United Kingdom. Eur J Clin Microbiol Infect Dis. 2000;19(11):834‐842. 11152308 10.1007/s100960000376

[5] Hirve S , Newman LP , Paget J , et al. Influenza seasonality in the tropics and subtropics—When to vaccinate? PLoS One. 2016;11(4):e0153003. 27119988 10.1371/journal.pone.0153003 PMC4847850

[6] Emukule GO , Mott JA , Spreeuwenberg P , et al. Influenza activity in Kenya, 2007–2013: Timing, association with climatic factors, and implications for vaccination campaigns. Influenza Other Respi Viruses. 2016;10(5):375‐385. 10.1111/irv.12393 PMC4947939 27100128

[7] Rafiq D , Batool A , Bazaz MA . Three months of COVID‐19: A systematic review and meta‐analysis. Rev Med Virol. 2020;30(4):e2113. 32420674 10.1002/rmv.2113 PMC7267122

[8] Radin JM , Katz MA , Tempia S , et al. Influenza surveillance in 15 countries in Africa, 2006‐2010. J Infect Dis. 2012;206(1):2006‐2010. 10.1093/infdis/jis606 23169960

[9] WHO . WHO epidemiological influenza surveillance standards. 2014:2–4.

[10] Katz MA , Muthoka P , Emukule GO , et al. Results from the first six years of national sentinel surveillance for influenza in Kenya, July 2007–June 2013. PLoS One. 2014;9(6). 10.1371/journal.pone.0098615 PMC4067481 24955962

[11] Lina B , Georges A , Burtseva E , et al. Complicated hospitalization due to influenza: Results from the Global Hospital Influenza Network for the 2017‐2018 season. BMC Infect Dis. 2020;20(1):1‐14. 10.1186/s12879-020-05167-4 PMC7330273 32615985

[12] Dutta A , Mainia T , Ginivan M & Koseki S. Kenya health financing system assessment. 2018.

[13] Health MOF . Kenya Household Health Expenditure and. 2014;(December).

[14] Andrew MK , McElhaney JE , McGeer AA , et al. Influenza surveillance case definitions miss a substantial proportion of older adults hospitalized with laboratory‐confirmed influenza: A report from the Canadian Immunization Research Network (CIRN) Serious Outcomes Surveillance (SOS) Network. Infect Control Hosp Epidemiol. 2020;41(5):499‐504. 32146920 10.1017/ice.2020.22

[15] Nyamusore J , Rukelibuga J , Mutagoma M , et al. The national burden of influenza‐associated severe acute respiratory illness hospitalization in Rwanda, 2012–2014. Influenza Other Respi Viruses. 2018;(August 2017);12(1):38‐45. 10.1111/irv.12494 PMC5818355 29197152

[16] Theo A , Tempia S , Cohen AL , et al. The national burden of influenza‐associated severe acute respiratory illness hospitalization in Zambia, 2011‐2014. Influenza Other Respi Viruses. 2018;12(1):46‐53. 10.1111/irv.12492 PMC5818337 29243406

[17] Peterson I , Bar‐Zeev N , Kennedy N , et al. Respiratory virus‐associated severe acute respiratory illness and viral clustering in Malawian children in a setting with a high prevalence of HIV infection, malaria, and malnutrition. J Infect Dis. 2016;214(11):1700‐1711. 27630199 10.1093/infdis/jiw426 PMC5341080

[18] Abadom TR , Smith AD , Tempia S , Madhi SA , Cohen C , Cohen AL . Risk factors associated with hospitalisation for influenza‐associated severe acute respiratory illness in South Africa: A case‐population study. Vaccine. 2016;34(46):5649‐5655. 27720448 10.1016/j.vaccine.2016.09.011 PMC5714809

[19] WHO_Europe _ 2019–2020 influenza season_ repurposing surveillance systems for COVID‐19.

[20] Africa S , Cohen C , Moyes J , Tempia S , Groome M. Mortality amongst patients with influenza‐associated severe acute respiratory illness. 2015:2009‐2013. 10.1371/journal.pone.0118884 PMC4365037 25786103

[21] Kandeel A , Dawson P , Labib M , Said M , El‐refai S , Morbidity E‐A . Mortality, and seasonality of influenza hospitalizations in Egypt, November 2007–November 2014. 2016;11(9):1‐14. 10.1371/journal.pone.0161301 PMC5015910 27607330

[22] McMorrow ML , Wemakoy EO , Tshilobo JK , et al. Severe acute respiratory illness deaths in Sub‐Saharan Africa and the role of influenza: A case series from 8 countries. J Infect Dis. 2015;212(6):853‐860. 25712970 10.1093/infdis/jiv100 PMC4826902

[23] Macias AE , Precioso AR , Falsey AR . The global influenza Initiative recommendations for the vaccination of pregnant women against seasonal influenza. Influenza Other Respi Viruses. 2015;9(S1):31‐37. 10.1111/irv.12320 PMC4549100 26256293

[24] Cohen C , Moyes J , Tempia S , et al. Severe influenza‐associated respiratory infection in high HIV prevalence setting, South Africa, 2009‐2011. Emerg Infect Dis. 2013;19(11):1766‐1774. 24209781 10.3201/eid1911.130546 PMC3837669

[25] Tempia S , Walaza S , Moyes J , et al. Risk factors for influenza‐associated severe acute respiratory illness hospitalization in South Africa, 2012–2015. Open Forum Infect Dis. 2017;4(1). 10.1093/ofid/ofw262 PMC5414019 28480255

[26] Diop D , Montomoli E , Melvin Sanicas MD . Influenza‐associated morbidity and mortality in Sub‐Saharan Africa. Int J Vaccines Vaccin. 2016;2(4):2‐7.

[27] Pannaraj PS , Wang H , Rivas H , et al. School‐located influenza vaccination decreases laboratory‐confirmed influenza and improves school attendance. Clin Infect Dis. 2014;59(3):325‐332. 24829215 10.1093/cid/ciu340 PMC4155443

[28] Herbert NL , Gargano LM , Painter JE , et al. Understanding reasons for participating in a school‐based influenza vaccination program and decision‐making dynamics among adolescents and parents. Health Educ Res. 2013;28(4):663‐672. 23727620 10.1093/her/cyt060 PMC3708138

[29] Baguelin M , Flasche S , Camacho A , Demiris N , Miller E , Edmunds WJ . Assessing optimal target populations for influenza vaccination programmes: An evidence synthesis and modelling study. 2013;10(10):e1001527. 10.1371/journal.pmed.1001527 PMC3793005 24115913

[30] Dawa JA , Chaves SS , Nyawanda B , et al. National burden of hospitalized and non‐hospitalized influenza‐associated severe acute respiratory illness in Kenya, 2012‐2014. Influenza Other Respi Viruses. 2018;12(1):30‐37. 10.1111/irv.12488 PMC5818348 29243402

