# Supplementary material for: Severe acute respiratory illness surveillance for influenza in Kenya: Patient characteristics and lessons learnt
Source: Influenza Other Respir Viruses. 2022 Mar 14;16(4):740–8. doi: 10.1111/irv.12979 (PMC9111565; doi:10.1111/irv.12979)
Supplement: Supplementary file 1 — Figure S1. Distribution of influenza virus types and subtypes detected among children with severe acute respiratory illness by month, 2014–2018, Kenya (N = 716) [file IRV-16-740-s002.docx]

**Supplemental Figure 1. Distribution of influenza virus types and subtypes detected among children with severe acute respiratory illness by month, 2014-2018, Kenya (N=716)**
