# Supplementary material for: Severe acute respiratory illness surveillance for influenza in Kenya: Patient characteristics and lessons learnt
Source: Influenza Other Respir Viruses. 2022 Mar 14;16(4):740–8. doi: 10.1111/irv.12979 (PMC9111565; doi:10.1111/irv.12979)
Supplement: Supplementary file 2 — Table S1. Case Fatality Percentages (CFP) of children admitted with severe respiratory acute illness (SARI), 2014–2018, Kenya [file IRV-16-740-s001.docx]

**Supplemental Table 1. Case Fatality Percentages (CFP) of children admitted with severe respiratory acute illness (SARI), 2014-2018, Kenya**

| **Characteristic** | **Influenza positive** | **Deaths** | **CFP (95%CI)** | **Influenza negative** | **Deaths** | **CFP (95%CI)** | **P value** |
| --- | --- | --- | --- | --- | --- | --- | --- |
| **Age group** | 716 | 23 | 3.2 (2.1‒4.8) | 6287 | 217 | 3.5 (3.0‒3.9) | 0.674 |
| <6months | 68 | 6 | 8.8 (2.1‒15.6) | 1326 | 62 | 4.7 (3.5‒5.8) | 0.121 |
| 6‒23months | 357 | 14 | 3.9 (2.2‒6.5) | 3344 | 125 | 3.7 (3.1‒4.4) | 0.865 |
| 2‒<5years | 224 | 3 | 1.3 (0.3‒3.9) | 1266 | 19 | 1.5 (0.9‒2.3) | 0.857 |
| 5‒17years | 67 | 0 | 0 | 351 | 11 | 3.1 (1.6‒5.5) | 0.142 |
| **Sex** |  |  |  |  |  |  |  |
| Males | 393 | 15 | 3.8 (2.2‒6.2) | 3529 | 120 | 3.4 (2.8‒4.1) | 0.667 |
| Females | 323 | 8 | 2.5 (1.1‒4.8) | 2758 | 97 | 3.5 (2.9‒4.3) | 0.332 |
| **Time from illness onset to hospitalization** |  |  |  |  |  |  |  |
| 0‒3 | 446 | 13 | 2.9 (1.6‒4.9) | 4261 | 113 | 2.7 (2.2‒3.2) | 0.741 |
| 4‒7 | 217 | 5 | 2.3 (0.8‒5.3) | 1670 | 83 | 4.9 (4.0‒6.1) | 0.080 |
| >7 | 42 | 5 | 11.9 (3.9‒25.6) | 265 | 20 | 7.6 (4.7‒11.4) | 0.337 |
| **Length of hospital stay** |  |  |  |  |  |  |  |
| 0‒3 | 253 | 7 | 2.8 (1.1‒5.6) | 2188 | 109 | 5.0 (4.1‒6.0) | 0.116 |
| 4‒7 | 251 | 8 | 3.2 (1.4‒6.2) | 2125 | 56 | 2.6 (2.0‒3.4) | 0.610 |
| >7 | 175 | 8 | 4.6 (2.0‒8.8) | 1460 | 50 | 3.4 (2.6‒4.5) | 0.441 |
| **Co-morbidities** |  |  |  |  |  |  |  |
| Underlying illness^a^ | 139 | 6 | 4.3 (1.6‒9.2) | 1250 | 86 | 6.9 (5.5‒8.4) | 0.250 |
| Tested HIV^b^ | 2 | 0 | 0.0 | 19 | 3 | 15.8 (3.4‒39.6) | 0.542 |
| Tested malaria^c^ | 32 | 0 | 0.0 | 222 | 6 | 2.7 (1.0‒5.8) | 0.347 |
| Tuberculosis treatment | 5 | 1 | 20.0 (0.5‒71.6) | 79 | 13 | 16.5 (9.1‒26.5) | 0.834 |

^a^Includes: chronic respiratory illness, chronic neuromuscular or neurological disease, newly diagnosed tuberculosis, HIV/AIDS, chronic cardiac, liver or renal disease, malnutrition, diabetes, asthma or cancer, sickle cell disease, rickets

^b^Number of positive HIV cases out of 1,140 SARI cases tested during hospitalization

^c^Number of malaria positive cases out of 1,276 SARI cases tested during hospitalization
